# Quantitative assessment of silicone oil release with siliconized and silicone oil-free syringes by microflow imaging microscopy

**DOI:** 10.5935/0004-2749.2021-0320

**Published:** 2023

**Authors:** Lydianne Lumack do Monte Agra, Natasha Ferreira Santos da Cruz, Vaida Linkuviene, John F. Carpenter, Michel Eid Farah, Gustavo Barreto Melo, Maurício Maia

**Affiliations:** 1 Hospital de Olhos de Sergipe, Aracaju, SE, Brazil; 2 Universidade Federal de Sergipe, São Cristovão, SE, Brazil; 3 Department of Ophthalmology, Escola Paulista de Medicina, Universidade Federal de São Paulo, São Paulo, SP, Brazil; 4 Department of Pharmaceutical Sciences, University of Colorado, Anschutz Medical Campus, CO, USA

**Keywords:** Syringes, Intravitreal injection, Bevacizumab, Silicone oil, Seringas, Injeção intravítrea, Oleo de silicone, Bevacizumab

## Abstract

**Purpose:**

Since particles are released in syringes during intravitreal injections, we assessed them quantitatively after agitating syringes commonly used for intravitreal injections.

**Methods:**

With and without agitation, the SR 1-ml insulin, Becton-Dickinson Ultra-Fine 0.3-ml Short Needle with a half-unit scale, HSW Norm-Ject Tuberculin, and Becton-Di­ckinson 1-ml Luer Lok Tip were examined with buffer and bevacizumab, aflibercept, and ziv-aflibercept. Flow imaging microscopy was performed to assess the particle numbers, concentrations, morphology, and size distribution.

**Results:**

Using the Becton-Dickinson Ultra-Fine syringe, the average particle count after agitation was higher than in the no-agitation group. For particles greater than 10 and 25 µm, differences were observed using the SR syringe between the two studied conditions. There were no significant differences in the means for the other syringes. Without agitation, the SR syringe had the highest number of particles (2,417,361.7 ± 3,421,575.5) followed by the Becton-Dickinson Ultra-Fine with 812.530,9 ± 996.187,2. The Becton-Dickinson Luer Lok Tip and HSW Norm-Ject performed equally with 398,396.8 ± 484,239.2 and 416,016.4 ± 242,650.1 particles, respectively.

**Conclusions:**

Flicking syringes to eliminate air bubbles results in increased numbers of particles released during intravitreal injections into the human vitreous.

## Abstract (pt)

**Objetivo:**

Visto que partículas são liberadas nas seringas durante as injeções intravítreas (IVIs), estas foram avaliadas quantitativamente após a agitação das seringas mais comumente usadas para injeções intravítreas.

**Métodos:**

A seringa SR de 1 ml de insulina, a agulha curta Becton-Dickinson Ultra-Fine 0,3 ml com escala de meia unidade, HSW Norm-Ject Tuberculin e a Becton-Dickinson Luer Lok Tip de 1 ml foram estudadas com placedo e com bevacizumabe, aflibercept e ziv-aflibercept, com e sem agitação. MicroFlow Imaging Mi­croscopy foi realizada para avaliar o número de partículas, con­centração, morfologia e distribuição das mesmas por tamanho.

**Resultados:**

A contagem média de partículas após agitação foi maior do que no grupo sem agitação usando a seringa Becton-Di­ckinson Ultra-Fine. Diferenças foram observadas usando a seringa SR entre as duas condições estudadas para partículas maiores que 10 e 25 µm. Para as demais seringas, não foram observadas diferenças significativas nas médias. A seringa SR apresentou o maior número de partículas sem agitação (2.417.361,7 ± 3.421.575,5) seguida da Becton-Dickinson Ultra-Fine com 812.530,9 ± 996.187,2. A BD Luer Lok Tip e a HSW Norm-Ject se comportaram de forma semelhante com 398.396,8 ± 484.239,2 e 416.016,4 ± 242.650,1 partículas, respectivamente.

**Conclusões:**

Agitar seringas para remover bolhas de ar resulta em um maior número de partículas liberadas durante Becton-Dickinson no vítreo humano.

## INTRODUCTION

Almost three decades ago, protein particle formation was evaluated and associated with the presence of silicone oil (SO) in the insulin after analyzing a “cloudy” insulin formulation administrated with plastic syringes in patients with uncontrolled diabetes. This was SO’s first report of contaminated protein formulations^(1)^. Numerous reports were published in 2016 about SO droplets in patients’ vitreous cavities following intravitreal injections (IVIs) with insulin syringes, prompting the American Society of Retina Specialists to issue a member alert about SO droplets from the insulin syringes commonly used during those procedures^(2,3)^.

Since then, studies have been conducted to assess the SO released by syringes as well as the detection of these droplets in the vitreous. Bakri and Ekdawi and Khurana et al. estimated that the presumed SO droplets in the vitreous following IVIs ranged from 0.03% to 1.7% of the performed IVI^(4,5)^. Melo et al. and others researchers found SO droplets in the vitreous of 68% and 75% of eyes in the same condition, respectively, when examined by slit-lamp and ultrasonography, respectively^(4-7)^. Thompson reported SO droplets in the vitreous in 78% of eyes treated with bevacizumab (Avastin^©^, Genentech Inc., South San Francisco, CA) IVIs^(8)^.

Despite the long-term use of SO during vitreoretinal surgeries and the fact that it is considered safe for use in the eyes, many patients with SO droplets in the vitreous have persistent complaints of floaters, which may necessitate surgical intervention in extreme cases, as well as long-term elevation of intraocular pressure and severe uveitis^(9,10)^.

SO droplets and their aggregates may negatively impact formulation safety and stability. Injecting therapeutic proteins into SO droplets may stimulate immunogenicity in patients triggered by subvisible particles comprised of isolated proteins and proteins with particulate contaminants, such as air bubbles, fibers, glass, metal, and especially SO^(11-14)^. Chisholm et al. demonstrated that the presence of SO microdroplets in protein formulations might cause structural alterations in those proteins and create aggregates that might lead to an autoimmune response in mice. The group also showed that the concentration of SO utilized as a lubricant in the syringes, as well as the concentration of particles, directly affected the degree of the antibody response^(15)^.

Another factor is the amount of agitation that the formulations are subjected to. In laboratory studies, a synergistic effect that may result from combining the SO with the therapeutic protein solution by agitating the syringe has been observed, and the interactions between SO/water interfaces, air/water interfaces, and agitation seem to negatively affect the protein formulations, although the mechanism remains uncertain^(16,17)^. Melo et al. assessed the most commonly used syringes during this procedure after clinical observation of a substantial number of SO droplets in the vitreous of patients who underwent IVIs and found that SO release happens routinely and even more frequently when the syringes are agitated by flicking^(18,19)^.

In light of these findings, the purpose of this study was to quantitatively assess the particle release after agitation of different syringe models commonly used during IVIs worldwide, to compare this release between different syringe models, different drugs, and the two different conditions (flicked and non-flicked syringes).

## METHODS

### Materials

Four syringes were used: SR 1 ml insulin (Saldanha-Ro­drigues, Pedro Juan Caballero, Paraguay), Becton-Di­ckinson (BD) Ultra-Fine 0.3-ml Short Needle with a half-unit scale (Becton-Dickinson and Co., Franklin Lakes, NJ), HSW Norm-Ject Tuberculin (Henke Sass Wolf, Tuttlingen, Germany), and BD 1 ml Luer Lok Tip. All syringes were studied with a buffer and 3 three drugs: bevacizumab (Avastin^®^, Roche Diagnostics, Indianapolis, IN), aflibercept (Eylea^®^, Regeneron Pharmaceuticals, Tarrytown, NY), and ziv-Aflibercept (Zaltrap^®^, Sanofi-Aven­tis, Bridgewater, NJ). The syringes were evaluated with and without agitation by flicking. The buffer formulation (composition: 300 g of α-trehalose dihydrate, 2.9 g of sodium phosphate [monobasic, monohydrate], 0.6 g of sodium phosphate [dibasic, anhydrous], 0.2 g of polysorbate 20, water for injection [for 500 ml buffer] at pH 6.2) was provided by Vaida Linkuviene (Skaggs School of Pharmacy and Pharmaceutical Sciences, University of Colorado Anschultz, Aurora, CO). Bevacizumab, afli­bercept, and ziv-aflibercept were purchased from pharmacies for the experiments.

### Syringe preparation

For each sample, 50 µL of the fluid (drug or buffer) was aspirated with the syringes using appropriate needle attachment and expelled into an Eppendorf tube containing 950 µl of the buffer, thus comprising 1 ml of the sample. In the agitation group, the syringes with 50 µl of the fluid were flicked 15 times with the finger in a standardized fashion before loading the fluid into the Eppendorf tube; in the non-agitation group, the syringes were handled gently without flicking and the sample was loaded into the Eppendorf tube for analysis by microflow imaging microscopy. All experiments were performed by a single operator. In both the groups, a 25-gage BD Precision Glide needle was attached to the syringes to avoid fluid leakage from the syringe tip during the subsequent agitation and handling. The BD Ultra-Fine syringe was an exception because it has its own staked-in needle.

### Microflow imaging microscopy

The subvisible particles were enumerated and the concentration, morphology, and size distributions were assessed by flow imaging microscopy (Flowcam Fluid Imaging Technologies, Scarborough, ME) using the following settings: flow rate, 0.15 ml/min; autoimage rate, 25 frames/s; and flow cell type FC80FV. Purified water was used to clean the equipment before each experiment, and the background count was checked before every sample measurement. The presence of <50 particles/ml was considered an acceptable background value. All measurements were conducted in triplicate.

### Statistical analysis

The quantities of SO were analyzed descriptively and expressed as the mean and standard deviation. The Mann-Whitney non-parametric test was applied to compare the means based on the agitation conditions (agitation or no agitation) owing to the limited sample size (<10/group). For all statistical tests, a significance level of 5% was set. Statistical analyzes were performed using the STATA 12 software (StataCorp, 2011, Stata Statistical Software: Release 12. College Station, TX: StataCorp LP).

## RESULTS

Ninety-six syringe samples were assessed, including 24 units from each brand. For all syringes and particle sizes in the BD Ultra-Fine syringe, the average particle count released after agitation was higher than in the no-agitation group (Table 1). The BD Ultra-Fine syringe had 812,530.9 ± 996,187.2 particles in the no-agitation group versus 3,969,069.2 ± 4,021,052.6 in the agitation group (p=0.002). For the SR syringe, differences were observed between the two studied conditions for particles greater than 10 µm (p=0.015) and 25 µm (p=0.006). For the other syringes (HSW Norm-Ject and BD Luer Lok Tip), no significant differences in the means were seen in the total number of particles or the size distribution.

**Table 1:** Mean and standard deviation of silicone oil released during agitation, in accordance with the particle size and syringe type

**Size (um)**	**N**	**No agitation**	**N**	**Agitation**	**p-value**
		**Mean ± SD**		**Mean ± SD**	
BD Ultra-Fine	12	812,530.9 ± 996,187.2	12	3,969,069.2 ± 4,021,052.6	0.002
>1	12	414,590.0 ± 492.385,8	12	1,992,256.1 ± 2,047,597.7	0.003
>2	12	292,156.4 ± 357,694.0	12	1,434,576.1 ± 1,457,525.6	0.002
>5	12	87,775.5 ± 120,940.4	12	450,137.8 ± 448,722.0	0.001
>10	12	17,088.7 ± 25,699.1	12	88,273.3 ± 97,635.4	0.003
>25	12	920.3 ± 1,284.1	12	3,825.8 ± 4,334.7	0.006
BD Luer Lok	12	398,396.8 ± 484,239.2	12	409,283.7 ± 411,409.2	0.954
>1	12	227,456.1 ± 256,820.0	12	193,763.3 ± 155,755.3	0.908
>2	12	138,593.9 ± 179,309.5	12	1,266,21.7 ± 106,571.5	0.954
>5	12	27,235.0 ± 45,561.1	12	43,530.6 ± 64,051.6	0.564
>10	12	4,130.2 ± 5,545.1	12	25,161.1 ± 59,172.0	0.419
>25	12	981.6 ± 1,692.6	12	20,207.1 ± 58,356.3	0.371
HSW Norm-Ject	12	416,016.4 ± 242,650.1	12	517,335.8 ± 442,003.7	0.817
>1	12	252,913.9 ± 153,283.0	12	314,298.9 ± 279,087.3	0.817
>2	12	140,315.6 ± 80,986.2	12	173,217.8 ± 140,324.8	0.773
>5	12	19,940.0 ± 10,814.2	12	25,743.3 ± 19,998.4	0.603
>10	12	2,155.8 ± 1,822.3	12	3,186.7 ± 3,080.7	0.386
>25	12	691.1 ± 698.9	12	889.2 ± 1,061.4	0.729
SR	12	2,417,361.7 ± 3,421,575.5	12	4,064,340.0 ± 3,612,030.5	0.106
>1	12	1,270,677.8 ± 1,798,480.8	12	2,070,670.6 ± 1,846,307.7	0.119
>2	12	870,911.1 ± 1,250,551.4	12	1,466,197.2 ± 1,323,931.0	0.119
>5	12	232,994.4 ± 323,809.8	12	432,095.6 ± 385,764.3	0.083
>10	12	40,423.3 ± 54,580.4	12	86,735.0 ± 70,889.3	0.015
>25	12	2,355.0 ± 2,586.3	12	8,641.7 ± 8,612.5	0.006

P-value by the Mann-Whitney test. N= number; SD= standard deviation.

**Figure 1:**
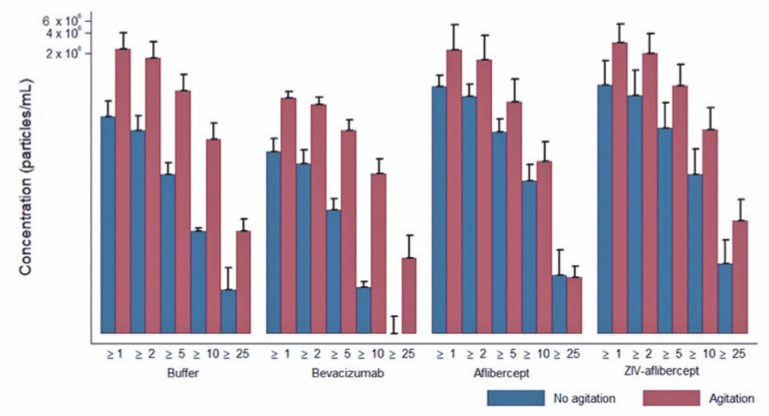
Average and standard deviation of particle released during agitation condition, in accordance to the solution and particle size for the BD Ultra-Fine. Media ± DP. Axis of ordinates on a logarithmic scale.

**Figure 2:**
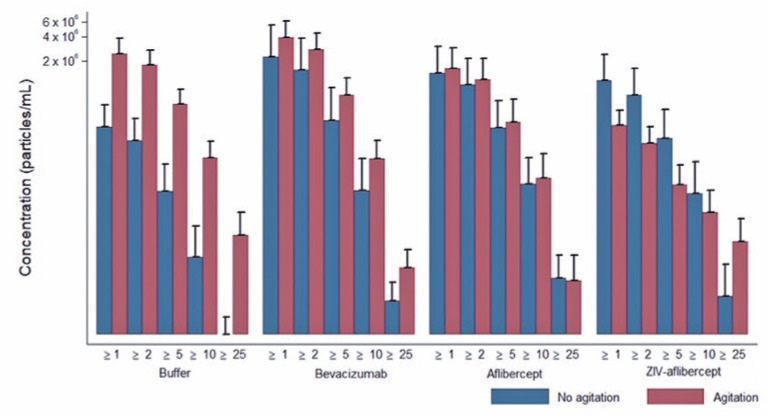
Average and standard deviation of particles released during agitation condition, according to the solution and particle size of the SR syringe. Media ± DP. Axis of the ordinates on a logarithmic scale.

**Figure 3:**
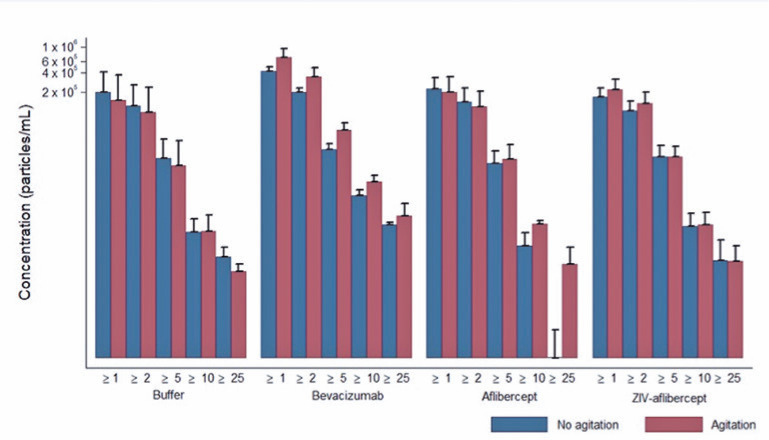
Average and standard deviation of the particles released during agitation, according to the solution and particle size for the HSW Norm-Ject. Media ± DP. Axis of the ordinates on a logarithmic scale.

**Figure 4:**
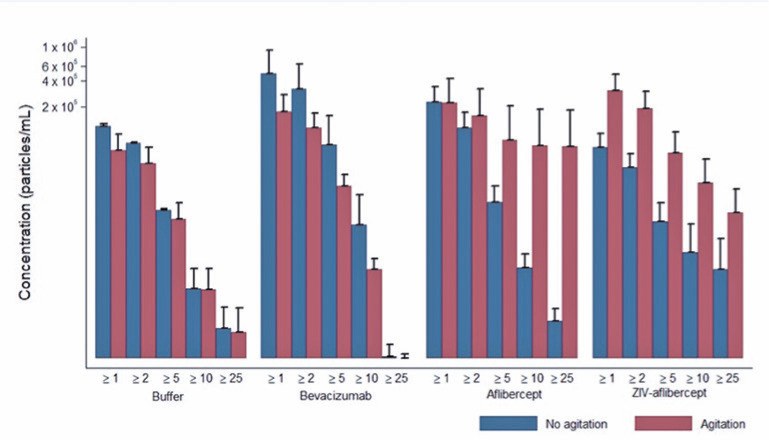
Average and standard deviation of particles released during agitation, according to the solution and particle size of the Luer Lok Tip. Media ± DP. Axis of the ordinates on a logarithmic scale.

There were no significant differences between the syringes when the buffer and three drugs were included, indicating that the drugs do not seem to be involved in particle release (Figures 1, 2, 3, and 4).

The SR syringe had the highest number of particles without agitation (2,417,361.7 ± 3,421,575.5) followed by the BD Ultra-Fine with 812.530,9 ± 996.187,2. The BD Luer Lok Tip and HSW Norm-Ject behaved similarly with 398,396.8 ± 484,239.2 and 416,016.4 ± 242,650.1, respectively.

## DISCUSSION

The main objectives of using SO as a lubricant for needles and syringes are to facilitate smooth sliding across surfaces, eliminate loose breakage, and minimize patient pain and injury to the ocular tissues. However, this same SO is seen in the vitreous of patients undergoing IVIs, and its clinical consequences are being studied^(4)^. The agitation of the syringes during transportation or, notably, during physician handling is one of the variables that might lead to a higher release of SO droplets^(1)^.

The BD Ultra-Fine 0.3 ml, the only syringe in this study with a fixed needle, was associated with a significantly higher particle concentration following agitation, between the two studied groups with and without agitation. This syringe’s potential to release particles was reported previously and flicking these syringes significantly increases the release^(7)^. The small dead space that characterizes syringes with fixed needles contributes to reduced particle release since the particles can remain in the dead space while the fluid is ejected from the syringe due to its lower viscosity ^(20)^. However, in the current study, the opposite was found. The two syringes with dead spaces, the BD Ultra-Fine and the SR 1-ml, released more particles before and after flicking, possibly due to the size of the studied particles, that is, larger particles (over 100 µm and visible to the naked eye) could be trapped in the dead space, which was not seen with smaller particles and evaluated in this study.

**Figure 5:**
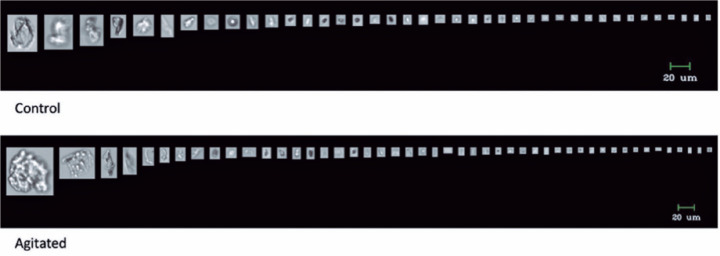
The **i**mage of the released particles in the control and agitated groups using the BD Ultra-fine syringe.

The SR 1-ml insulin, like the BD Ultra-Fine, was asso­ciated with a marked increase in particles released, including SO, following agitation, especially particles greater than 10 and 25 µm, despite the fact those syringes did not have staked-in needles. Figure 5 depicts particles released in the two groups: with and without agitation. The morphological difference between the two groups may be recognized.

**Figure 6:**
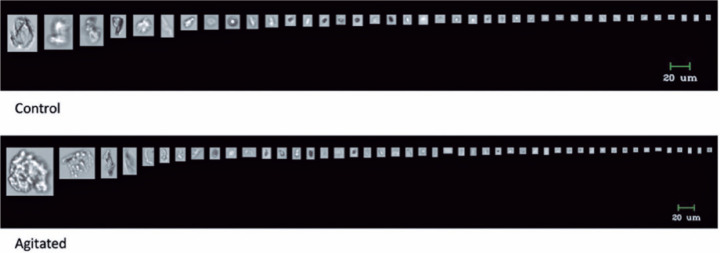
An image of the released particles in the control and agitated groups with the HSW syringe.

The HSW Norm-Ject, an oil-free syringe, and BD 1-ml Luer Lok Tip syringes did not significantly increase particle release following agitation. The number of particles released was low in the non-agitation group, and we hypothesize that the particles were originated from the buffer and drugs used in the study, as well as from the needle, rather than from the syringes *per se.* To avoid the adverse effects of SO, the manufacturer of the oil-free syringe has in some cases used alternative lubricants, such as oleamide, which may be a possible source of the particles in this study^(21)^. Figure 6 depicts an image of the particles released by the HSW syringe.

It is necessary to emphasize that even when the increase in released particles was not significant, there was an unequivocal increase in the number of particles after agitation, and the clinical impact of the presence of SO, fibers, and other particles in the human eye is still being investigated. Moreover, in addition to the consequences of particle release, any human tissue can have adverse side effects, such as the formation of skin granulomas resulting from a reaction in the subcutaneous tissue, triggering of a pulmonary embolus resulting from foreign particles in the bloodstream, or an autoimmune/inflammatory syndrome induced by adjuvants^(22,23)^.

The research is focusing on the possible clinical implications of SO and other particles in the medication and the human eye, i.e., not only the patient complaints and their consequent repercussions, but also the interactions of the particles with the drugs, formation of protein aggregates, and the immunogenic reactions resulting from the interaction of these aggregates, all of which are important because of the potential to compromise the efficacy and safety of the main medications currently used.

The study had some limitations, such as the small number of syringe models (four brands, one oil-free), the fact that one syringe (BD Ultra-Fine) had a staked-in needle and was not studied with the standard needle, and the technical limitation of microflow imaging microscopy, which cannot count the SO droplets separately from the other particles.

Users of these syringes have observed particle release for several years, and this study quantified this release as well as the effect of agitation on this process. Flicking syringes to remove air bubbles, a standard procedure among IVIs surgeons is one of the most important factors that enhances particle release into the human vitreous, even when the increase is insignificant.
